# Editorial: Physical exercise for age-related neuromusculoskeletal disorders

**DOI:** 10.3389/fnagi.2022.1099417

**Published:** 2022-12-16

**Authors:** Yuchen Wang, Min Hu, Li Li, Dongsheng Xu, Howe Liu, Xueqiang Wang, Kailimi Li

**Affiliations:** ^1^Department of Sport Rehabilitation, Shanghai University of Sport, Shanghai, China; ^2^Guangzhou Sport University, Guangzhou, China; ^3^Georgia Southern University, Statesboro, GA, United States; ^4^Shanghai University of Traditional Chinese Medicine, Shanghai, China; ^5^University of North Texas Health Science Center, Fort Worth, TX, United States

**Keywords:** physical exercise, aging, neurodegenerative disorders, musculoskeletal degenerative disorders, mechanism

Age-related neurological and musculoskeletal disorders with increasing life expectancy are emerging as major public health problems. Neurological disorders occur when neurons in the brain and spinal cord begin to degenerate such as Alzheimer's disease, Parkinson's disease, Huntington's disease, multiple sclerosis, spinal stenosis, and amyotrophic lateral sclerosis. In addition, both motor and sensory peripheral nervous pathologies could negatively affect physical activity in the aging population. Meanwhile, musculoskeletal disorders are pathological conditions that affect muscle, bone, cartilage, joint, and connective tissue, thereby leading to physical and functional impairments in patients, including but not limited to, osteoarthritis (OA), intervertebral disc degeneration, rheumatoid arthritis, osteoporosis, sarcopenia, and ankylosing spondylitis. Frailty syndrome, gait abnormality, and balance deficit that are commonly seen in older adults could be combined effects of neural and orthopedic dysfunction. Moreover, neuromusculoskeletal disorders have high rates of prevalence among the aging population and became the primary reason to seek interventions.

Physical exercise has been widely utilized as an effective preventive and interventional measure for patients with neurological and musculoskeletal problems by providing health benefits without side effects. A few key interventional studies have shown clinically meaningful improvements associated with physical exercises among individuals with Parkinson's disease and fibromyalgia, which indicate that further investigations are needed for other types of age-related neuromusculoskeletal disorders. In particular, research to explore the possible mechanisms of the potential beneficial effects of physical exercise is still in its infancy.

Therefore, this Research Topic aims to gather original research, review, and study protocol on physical exercise in the prevention and management of age-related neuromusculoskeletal disorders. We are interested in manuscripts that report the biomechanical, physiological, and psychological effects as well as the underlying mechanisms of physical exercise for age-related neuromusculoskeletal disorders.

A total of 35 original and review articles have passed the peer review and were finally published. Among these papers, three works explained bone health and osteoporosis, five articles explored muscle diseases, 16 works discussed neurodegenerative diseases, five explored age-related diseases, and other six studies about Chinese traditional exercise. Moreover, we hope this topic will gain new ideas and stimulate available methods for future work in the field. These papers can be categorized into the following sessions.

## Bone health and osteoarthritis

Osteoarthritis (OA) is a common chronic irreversible bone disease characterized by the degeneration of articular cartilage and secondary bone hyperplasia. Ning et al. analyzed the production and features of irisin and explained various exercises for irisin to explore the mechanism of osteoarthritis and provide new ideas for the prevention and treatment of osteoarthritis, which is a myohormone released primarily by skeletal muscle, and the synthesis and secretion are induced by exercise-induced muscle contraction to readers. Thus, understanding the importance of exercise in the fight against osteoarthritis will continue to present important references about the treatment and prevention of osteoarthritis. Further studies about the effects of exercise forms and intensity on induced irisin expression, and the regulatory mechanisms of irisin on BMD and cartilage metabolism will implement preventive and therapeutic approaches for osteoarthritis. On that basis, a total of 51 studies were reviewed to elucidate the mechanism of physical activity alleviating the pathological changes of osteoarthritis (Kong H. et al.). Furthermore, they included 59 original articles to explore different types of exercise and parameters that may have different influences on patients with OA. The review expounded on exercise in the prevention and treatment of OA. The paper presented the exercise intervention to treat OA for future patients. Liu et al. conducted similar research that focused on the bone health problem of postmenopausal women.

## Muscle diseases

Animal research (Liang et al.) found that during exercise, skeletal muscle exhibits extensive metabolic and energetic remodeling. Thus, exploring the mechanism of exercise-induced acetylation changes in skeletal muscle is helpful to develop scientific exercise prescriptions. Hence, exercise is effective to treat muscle diseases such as sarcopenia, which is a common chronic disease with a loss in muscle strength, muscle mass, and physical abilities. The benefits of vibration training and resistance training on muscle function as a form of exercise have been confirmed by various previous studies. However, the comparison between vibration training and resistance training remains lacking in elderly patients with sarcopenia. In Lu's protocol (Lu et al.), this study conducted a 12-week, three-arm randomized controlled trial. A total of 54 participants were randomized into resistance training, vibration training, and control groups. Moreover, they compared the effect of vibration training and resistance training among old patients with sarcopenia on muscle strength, muscle mass, blood biomarkers, physical abilities, and life quality. Then, they added measurements of upper and lower limb muscle strength and blood biomarkers as compared to previous studies. Another systematic review by Guo et al. included 21 studies and 1,330 participants to explore the effect of traditional Chinese medicine on sarcopenia. They analyzed the present evidence for applying traditional Chinese medicine to treat sarcopenia. The study also suggested that traditional Chinese medicine has a significant effect on body function and muscle strength for people with sarcopenia.

Other types of muscle diseases can also be improved by exercise. Zhuang et al. analyzed 12 studies on physical activity (PA) effects in older people with sarcopenic obesity (SO). Aerobic training decreased body weight and body mass index; resistance training improved body fat, appendicular skeletal muscle mass index, appendicular skeletal muscle mass, handgrip strength, and knee extension strength as compared to a control group without PA. Meanwhile, the aerobic combined with resistance training improved body fat, body mass index, appendicular skeletal muscle mass index, handgrip strength, and PA increased insulin-like growth factor 1 (IGF-1) as compared to a control group without PA. The systematic review and meta-analysis showed that PA is an effective treatment to improve physical performance, muscle strength, muscle mass, body composition, and IGF-1 in SO elderly adults. Zhu et al. included 10 studies to perform a mixed comparison of different exercise interventions on function, respiration, fatigue, and quality of life in adults with amyotrophic lateral sclerosis (ALS). The study concluded that multimodal exercise and rehabilitation programs are more beneficial for patients with ALS. However, its safety and practice guidelines are unclear, which still need to be further confirmed by large samples and high-quality randomized controlled trials.

## Neurodegenerative diseases

Herein, neurodegenerative diseases such as stroke, Parkinson's disease (PD), movement disorders, multiple sclerosis, diabetic neuropathic pain, and Alzheimer's disease (AD) are included. Recent research trends in exercise therapy for some kind of neurodegenerative diseases were revealed by four bibliometric articles (Chen B. et al.; Chen J. W. et al.; Dong et al.; Jiang et al.) and provided potential research frontiers. This provided a useful basis for further research on priority issues, partners, and development trends.

Parkinson's disease is a kind of disease distinguished by bradykinesia, balance disruption, rigidity, and gait impairment. In an animal experimental study, 1-methyl-4-phenyl-1, 2, 3, 6-tetrahydropyridine (MPTP)-induced Parkinson's disease mice were used to observe their motor ability after 12-week treadmill training (Tong et al.). The results presented that a 12-week treadmill exercise dramatically improved the motor performance of the MPTP-induced PD mouse model, thereby suggesting that substantia nigra and striatum are essential to brain regions that undergo key transcriptional changes, after exercise intervention in the PD model. The effects of traditional Chinese exercise (TCE) on balance, gait outcomes, and motor symptoms in participants with PD were evaluated (Wu et al.). A total of 15 studies and 873 subjects were analyzed in this study. The results revealed significant improvements in balance outcomes and gait outcomes, and motor symptoms compared with control groups. This work presented that TCE is beneficial for motor symptoms, balance function, and gait function among patients with PD. Meanwhile, the additional study focused on aerobic and resistance training also contributed to this conclusion (Zhou et al.).

Stroke is the leading cause of death worldwide and will cause severe sensorimotor dysfunction and limitations. Li L. et al. explore immediate muscle electrical impedance property alterations among chronic stroke patients in the lower extremity instantly after functional electrical stimulation (FES)-assisted cycling training. This report stated that electrical impedance myography can make clear the changes in intrinsic characteristics of paralyzed muscles in chronic stroke patients after FES-assisted cycling training. In another work, 15 RCTs involving 488 participants were included (Li X. et al.). The combined data showed that after 3–12 weeks of intervention, the wearable sensor-based exercise group showed remarkable improvement as compared with the traditional exercise group. This study also showed a statistically significant difference in visual scores between post-assessment and 1-month follow-up assessment. In addition, this study indicated that wearable sensor-based exercise was advantageous in enhancing balance in people with neurodegenerative diseases, as evidenced by the Chen studies on bilateral arm training (Chen S. et al.).

Meanwhile, Alzheimer's disease is a common neurological disease and is the major cause of dementia worldwide. Li et al. researchers provided a comprehensive systematic review of combined exercise and music interventions in participants with Alzheimer's disease. They found that combined physical activity and music interventions are helpful to improve cognitive functioning and wellbeing among AD patients. The study concluded that physical and musical interventions are essential in assisting medical guidance by therapists. The combination of physical activity and music intervention improved gait disorders in PD patients with freezing of gait, thereby improving their comprehensive motor function (Li K.-P. et al.).

The effects of exercise on other different types of neurodegenerative diseases have also been studied (Liao Q. et al.; Luo et al.; Zhang Y. et al.). In addition, Zhang G. et al. included a total of 32 chronic back pain (CBP) patients without depressive symptoms and 30 CBP patients with depressive symptoms. All subjects underwent functional magnetic resonance imaging scans and clinical assessments of depressive symptoms and pain-related manifestations. The research concluded that comorbid depressive symptoms can aggravate the impairment of pain matrix function in CBP patients. However, this impairment cannot directly lead to an increase in pain intensity, which may be related to the fact that some brain regions of the pain matrix are the common neural basis of depression and CBP.

## Age-related diseases

Two reviews summarized previous studies showing that exercise can improve chronic pain in middle-aged and older adults. One presented by Wen et al. included 17 studies of the mind-body exercise (MBE) method in middle age people and senior citizens with chronic pain. It indicated that mind-body exercise had a modest effect on relieving pain compared with the non-active and active control group. The results suggested that MBE was a significant method for relieving chronic pain symptoms in middle-aged and older people as compared with the inactive and active groups. Another mini-review included a total of 11 randomized controlled trials with 1,256 middle-aged and elderly patients with chronic low back pain (CLBP) and concluded that traditional Chinese exercise can effectively relieve the pain of middle-aged and elderly patients with CLBP (Wang X.-Q. et al.).

In addition to the analgesic effects of exercise in middle-aged and older adults, another three studies examined whether or not exercise improves upper limb, cognitive, and executive functions in older adults. Meanwhile, Liao T. et al. examined a 12-week Wheelchair Tai Chi Ball (WTCB) treatment for older people with a physical disability. The results represented that the WTCB intervention had significant effects on the following domains: daily living activities, physical health, mental health, and maintaining upper limb muscle strength. The study concluded that WTCB could improve upper limb muscle strength in disabled older adults. Wang H. et al. researched to investigate the relationship between walking speed, cognitive impairment, and cognitive functions among elder people. The results showed that walking speed was inversely related to cognitive impairment in males. Apart from that, the relationship between walking speed and impaired orientation was noticeable for both men and women. Zheng et al.'s study about executive function ends up with the same conclusion.

## Traditional Chinese exercise

Tai Chi is one of the most common traditional Chinese exercises and has been widely used in clinical studies. Song et al. performed a study of 40 older females with knee osteoarthritis (KOA). In this study, subjects were grouped into a 12 weeks Tai Chi or control group. The results revealed that 12 weeks of Tai Chi was more effective to improve physical function and life quality in elder women with KOA as compared with the control group. Another study related to Tai Chi Chuan (TCC) was conducted (Huang et al.). Herein, a motion capture system was used to measure the three aspects of the motion of the pelvis and lower limbs in 15 elderly patients with TCC and 15 healthy controls. This work showed kinematic changes in the pelvic and lower limb joints and presented a strategy to decrease the risk of trips in long-term TCC trainers while crossing obstacles. Therefore, the protocol reported by Wang R. et al. aimed to conduct a single-blind randomized controlled trial involving older adults with chronic low back pain based on the two studies. They recruited a total of 138 participants and randomly assigned them in a 1:1 ratio (Tai Chi or physical therapy). Then, participants underwent a 40-week long-term impact study. Hence, this work demonstrated the feasibility and effectiveness of Tai Chi to treat low back pain. However, current studies and works on this topic have several limitations based on a comprehensive exploration. First, mechanisms of different physical exercises or activities for age-related neuromusculoskeletal disorders remained inadequate, because current works did not apply either large human samples or animal models. Consequently, their results were unreliable. Second, we did not find any related content about how to connect these basic physical exercises to clinical practice. Shao et al. also contributed to this conclusion.

As for other types of traditional Chinese exercises, in a review conducted by Kong L. et al., they included 21 studies related to neck pain to support the effectiveness of Traditional Chinese Exercises (TCEs). The combined results suggested that TCEs had positive effects on pain relief. Particularly, Baduanjin had a beneficial complementary effect on the improvement of neck flexion and extension. Furthermore, the combined results indicated that TCEs revealed positive influences in the improvement of disability and pain relief as compared with the waiting list. Another research focused on clinical efficacy and electroencephalogram signal characteristics of Yijinjing Qigong intervention on early post-stroke depression was also conducted by Sun et al..

However, there are still several limitations of current studies and works on this topic based on a comprehensive exploration. First, the mechanisms by which different physical exercises or activities contribute to age-related neuromusculoskeletal disorders remain inadequate, as current works do not apply to large human samples or animal models. Consequently, their results are not reliable. Second, we did not find any related content about how to connect these basic physical exercises to clinical practice.

Thus, we hope this work can inspire the interest of scholars researching the effects of physical exercise for age-related neuromusculoskeletal disorders, thereby improving future research studies with new strategies and high-quality design. Current experiments are still in the theoretical stage. Therefore, further work should pay more attention to these research frontiers.

## Author contributions

YW drafted the manuscript. KL reviewed the manuscript. All authors contributed to the article and approved the submitted version.

